# A new understanding of the cognitive reappraisal technique: an extension based on the schema theory

**DOI:** 10.3389/fnbeh.2023.1174585

**Published:** 2023-04-17

**Authors:** Ya-Xin Wang, Bin Yin

**Affiliations:** ^1^Laboratory of Learning and Behavioral Sciences, School of Psychology, Fujian Normal University, Fuzhou, Fujian, China; ^2^Department of Applied Psychology, School of Psychology, Fujian Normal University, Fuzhou, Fujian, China

**Keywords:** cognitive reappraisal, emotion regulation strategy, schema theory, schema updating, schema enrichment, habit formation, ecological efficacy

## Abstract

Cognitive reappraisal is a widely utilized emotion regulation strategy that involves altering the personal meaning of an emotional event to enhance attention to emotional responses. Despite its common use, individual differences in cognitive reappraisal techniques and the spontaneous recovery, renewal, and reinstatement of negative responses across varying contexts may limit its effectiveness. Furthermore, detached reappraisal could cause distress for clients. According to Gross’s theory, cognitive reappraisal is an effortless process that can occur spontaneously. When guided language triggers cognitive reappraisal as an emotion regulation strategy in laboratory or counseling settings, clients experience improved emotional states, but this induced strategy may not necessarily guide them in regulating emotions in similar future situations. Therefore, effectively applying cognitive reappraisal techniques in clinical practice to help clients alleviate emotional distress in daily life remains a significant concern. Exploring the mechanism of cognitive reappraisal reveals that reconstructing stimulus meaning is akin to extinction learning, which entails fostering cognitive contingency that the original stimulus provoking negative emotions will no longer result in negative outcomes in the current context. However, extinction learning is a new learning process rather than an elimination process. The activation of new learning relies on the presentation of critical cues, with contextual cues often playing a vital role, such as a safe laboratory or consulting room environment. We propose a new understanding of cognitive reappraisal based on the schema theory and the dual-system theory, emphasizing the significance of environmental interaction and feedback in constructing new experiences and updating schemata. This approach ultimately enriches the schema during training and integrates the new schema into long-term memory. Bottom-up behavioral experiences as schema enrichment training provide the foundation for top-down regulation to function. This method can assist clients in activating more suitable schemata probabilistically when encountering stimuli in real life, forming stable emotions, and achieving transfer and application across diverse contexts.

## 1. The present state of research and application of cognitive reappraisal

### 1.1. What is cognitive reappraisal

Cognitive reappraisal is a strategy for regulating emotions in which individuals reinterpret the meaning of stimuli to modify their emotional response (Gross, 1998b; Sheppes and Gross, 2011). Gross has classified emotion regulation strategies into two main categories based on factor analysis: cognitive reappraisal and expression suppression. Cognitive reappraisal is an antecedent-focused strategy of emotion regulation that takes place early in the emotional response process (An et al., 2015). Antecedent-focused strategies occur before the emotional responses are apparent and refer to cognitive alterations made in response to a situation (Appleton et al., 2014). Expression suppression, on the other hand, refers to the inhibition of emotional expression without modifying emotional experience and can increase emotional arousal (Gross, 1998a). A time course model of cognitive reappraisal and expression inhibition suggests that cognitive reappraisal occurs before emotions arise and does not require sustained cognitive effort or self-regulation (Goldin et al., 2008). Furthermore, cognitive reappraisal does not consume additional cognitive resources and therefore does not interfere with other cognitive activities (Ochsner and Gross, 2005). Appraisal theory also explains that emotions are triggered when individuals attend to and evaluate a situation as relevant to their currently active goals (Lazarus, 1991; Scherer et al., 2001; Gross, 2014). Therefore, cognitive reappraisal has certain benefits in regulating negative emotional reactions compared with expression suppression (Cheng et al., 2009; Bai et al., 2015).

### 1.2. Scenarios where cognitive reappraisal is used

In the context of emotion regulation, cognitive reappraisal is typically applied to help individuals recognize and correct maladaptive thoughts (Gross, 2014). Cognitive reappraisal is often presented in the form of conversation in the consultation room. For instance, in the book “Process-Based CBT: The Science and Core Clinical Competencies of Cognitive Behavioral Therapy” (Hayes and Hofmann, 2018), cognitive reappraisal techniques were used to help a client named Lisa recognize her core belief of “I’m not popular” and the assumption that “if a person is really a friend, she will invite me to important social activities” when she was not invited to a friend’s expectant mother’s party. The therapist helped Lisa assess the accuracy and usefulness of her thoughts and understand that it was reasonable to be disappointed about not being invited to the party, but that the friend’s extended family might have been prioritized, which did not indicate that the friend did not like Lisa.

However, it is important to note that cognitive reappraisal is distinct from positive thinking, as its goal is to achieve a balance between realistic acceptability and positive emotions (Hayes and Hofmann, 2018). While cognitive reappraisal achieved through conversation may be meaningful to others, it may not translate to the client’s real-life experiences due to lack of practice. For example, individuals may become so deeply engrossed in their internal experiences that it becomes challenging to distinguish between logical and emotional distress (Hayes and Hofmann, 2018), resulting in unfulfilled inner needs. Incomplete cognitive reappraisal may prove ineffective or even detrimental if it leads to a sense of unreality or lacks a factual basis (Ford and Troy, 2019). Only the reappraisal effects that are closely linked to the client’s real life experiences are replicable across positive and negative emotional states (Brockman et al., 2023).

Furthermore, there are individual differences in the ability to use cognitive reappraisal in daily life (Troy et al., 2010). The use of spontaneous cognitive reappraisal (SRE) in daily life varies among individuals, and it is unclear whether guidance provided in counseling sessions can help individuals activate SRE in their everyday experiences (Volokhov and Demaree, 2010; An et al., 2015). Therefore, it is worth exploring whether conversation-based cognitive reappraisal can modify this automated process. For example, some clients may become fixated on the need for reframing or engage in ceaseless reframing with no discernible relief (Hayes et al., 2012; Grecucci et al., 2016). During bottom-up processing, these individuals may not have been exposed to the source of information or stimulus that challenges the expectation of negative outcome, nor to the stimulus evaluation and motivation processing needed to correct the deviated thoughts, thus sustaining the reinforcing cycle of depressive symptoms (Forgas and George, 2001; Beevers, 2005). Indeed, inability to successfully employ reappraisal and its limited functionality even when successful are well-known challenges (Ford and Troy, 2019). Research conducted in laboratory contexts has revealed that after attempting cognitive reappraisal on standardized emotion induction, one-third of participants reported feeling worse than their natural responses, as measured by self-report and physiological indicators (Troy et al., 2010). In daily life, nearly half of individuals who tried to use cognitive reappraisal when faced with negative events rated their success on the scale as not at all successful or slightly successful (Ford et al., 2017). Moreover, for those who lack cognitive reappraisal skills, frequent attempts at this technique are associated with higher levels of individual depressive symptoms (Ford et al., 2017). The success of cognitive reappraisal is influenced by situational factors, and employing it in situations where success is unlikely could pose a cumulative risk to an individual’s physical and mental health (Ford and Troy, 2019).

In summary, cognitive reappraisal is a technique used to assist individuals in recognizing and correcting maladaptive thoughts. It is commonly used in cognitive behavioral therapy to help clients modify their belief systems, thereby reducing the intensity of physical responses or adjusting the constructed meaning of those responses to the current situation. However, cognitive reappraisal induced in laboratory and consulting settings may lack the necessary supporting materials and real-world applicability. During emotion regulation, individuals may become more conscious of these changes since they have a specific objective of regulating their emotions through cognitive changes or response modulation. However, there are individual differences in the ability to spontaneously use cognitive reappraisal in daily life. Additionally, it is important to note that incomplete cognitive reappraisal may prove ineffective or even detrimental if it leads to a sense of unreality or lacks a factual basis. Therefore, it is important to understand why cognitive reappraisal may not always be effective and how to upgrade the technique to enhance the efficacy.

### 1.3. Reasons for differences in the efficacy of cognitive reappraisal

The variability in the efficacy of cognitive reappraisal may stem from the fact that this technique is often facilitated in controlled settings, such as laboratories or counseling rooms, using verbal guidance. The generalizability of the effects of cognitive reappraisal to other contexts remains uncertain. Research conducted in laboratory settings has shown that personal and situational factors significantly influence the effectiveness of implementing cognitive reappraisal (Cohen Ben Simon et al., 2022). Contextual factors play a critical role in reappraisal efficacy (Ford and Troy, 2019).

According to Kirsch et al. (2004), even higher-order processes can be understood as simple stimulus-response associations that function as response sets, which automatically trigger a response when specific stimulus conditions are met. Stimulus-outcome and response-outcome associations can be viewed as stimulus sets that prepare organisms to perceive environmental stimuli in particular ways. The concept of set connects automatic conditional response processes and representational cognitive processes, allowing organisms to respond automatically when sensing their environment and encountering appropriate stimulus conditions (Kirsch and Lynn, 1999). In higher-order cognition, explicit expectation refers to the conscious acquisition of sets of stimuli and responses, while unconsciously acquired sets represent implicit expectation (Kirsch et al., 2004). Thus, cognitive reappraisal shares similarities with exposure-based extinction learning, which forms new (null) associations between the original conditioned stimulus (CS) and the aversive unconditioned stimulus (US) in a new environment (Bouton et al., 2006). This process maintains the original learning while adding new inhibitory learning that suppresses the initially learned behavior (Lovibond, 2004; Ciocchi et al., 2010).

In exposure-based extinction learning, the original stimulus that was paired with an aversive stimulus is repeatedly presented without the aversive stimulus, leading to new inhibitory learning about the stimulus. The Pavlovian inhibitory learning model suggests that while the original association remains intact, new inhibitory learning develops, indicating that the stimulus no longer predicts the aversive stimulus (Craske et al., 2018). Conditioned reflexes arise from past experiences where a CS in the original environment elicits a US, leading to unconditional and conditional reflexes (UR and CR) and provoking uncontrolled emotions. However, if an individual returns to the original environment, the association between the original CS and US may be renewed (Bouton and King, 1983; Bouton et al., 2006; Heald et al., 2023). Even in a new context, the original memory of association can be restored automatically over time (Bouton, 1993, 2023; Bouton et al., 2006). Moreover, the memory of the original association may be reinstated when re-experiencing the US, even in the absence of the CS (Rescorla and Heth, 1975). Addressing the renewal, reinstatement, and spontaneous recovery of the CS-US association in the original memory presents a significant challenge.

Like exposure-based therapy, cognitive reappraisal involves learning new relationships between environmental stimuli and expected outcomes through mechanisms of extinction and generalization (Lovibond, 2004). Changes in context may hinder the retrieval of specific features of the reappraisal prompt, preventing generalization across contexts and inducing forgetting of the inhibitory association itself, which weakens the efficacy of reappraisal. From another perspective, when the context is the most accurate predictor of the US, the context can serve as a CS in a CS-US association. When the original CS is reintroduced, it activates the context representation, which in turn activates the US representation due to the new context-US association (Bouton et al., 2006). In reality, during the process of new learning, the client’s focus on the consultation or laboratory environment amplifies the contextual element, making the context itself, as a secondary CS, more closely associated with the desired US than the original CS that the consultant helps the client reactivate and reappraise, which can result in an overshadowing effect (Bouton, 2016), dampening the efficacy of reappraisal.

From a neurobiological perspective, during extinction learning and expression, the hippocampus signals the ventromedial prefrontal cortex (vmPFC) to activate local inhibitory networks in the amygdala. This leads to the downregulation of fear reactions when the test context closely resembles the extinction context (Greco and Liberzon, 2016; Maren and Holmes, 2016; Craske et al., 2018). LeDoux (2022) suggests that the higher-order network involved in emotion regulation includes the lateral prefrontal cortex (PFC), encompassing the dorsal and ventral prefrontal cortex and the lateral frontal pole. The dorsolateral prefrontal cortex (DLPFC) is involved in the executive control of emotional expression and is more characteristic of higher-order emotion regulation processes (Schiller and Delgado, 2010; Battaglia et al., 2020). The ventrolateral prefrontal cortex (VLPFC) is often associated with control over stimulus selection (Disner et al., 2011). Cognitive reappraisal downregulates negative emotions through increased activation of medial and lateral PFCs and decreased activation of emotional arousal-related brain structures such as the amygdala and insula (Ochsner and Gross, 2005; Ochsner et al., 2012; Cutuli, 2014). Moreover, emotion regulation processes like cognitive reappraisal are often associated with neural oscillatory activity in frontal and temporal sites, with alpha band activity decreasing during emotion self-regulation programs that involve happy emotion induction training (Ippolito et al., 2022). Recent research indicates that the vmPFC not only activates during the extinction or reversal of previously acquired learning but also plays a crucial role in acquiring new learning (Schiller and Delgado, 2010; Battaglia et al., 2020). Therefore, the cues needed to activate new learning become particularly important, as new learning is often activated in laboratory or consulting room settings. Strategies to enhance extinction generalization include extinguishing multiple variations of the conditioned stimulus, manipulating attention during generalization stimulus extinction toward common features, and promoting forgetting of specific features of the extinction stimulus (Barry et al., 2017; Craske et al., 2018). Exposure to disconfirming experiences (conditioned stimulus without unconditioned stimulus) should lead to revising the knowledge stored in long-term memory so that behavior is controlled by this updated knowledge (Lovibond, 2004).

Finally, it is important to recognize that low-level processes are essential for higher-level processes, such as those associated with conscious awareness (Lovibond, 2004). Research has shown that some individuals may lack access to social connections and supportive external voices in their lives, highlighting the need to develop their own internal supportive voice through cognitive reappraisal, which may not be easily achieved through mere “telling” (Brockman et al., 2023). Retrieving the CS-US contingency from long-term memory generates an expectancy of the US, leading to the automatic activation of innate anticipatory behaviors appropriate for the US. The current form of conversation-based reappraisal techniques does not directly address issues in the original environment (Context A), and the new positive relationship formed in a different environment (Context B) may not impact the original memory and association between CS and US. Therefore, positive experiences in Context B cannot simply counteract negative experiences in Context A. It is crucial to investigate the neural and psychological mechanisms of emotion regulation to gain a deeper understanding of the principles behind cognitive reappraisal and to determine whether its effects are generalizable across different contexts.

## 2. Neural and psychological mechanisms of cognitive reappraisal

### 2.1. Insights from LeDoux’s “two-system” framework of fear and other emotions

As one heavily studied field, brain mechanisms involved in fear learning and extinction can provide useful insights for understanding emotion regulation. Previous studies suggest that the amygdala is the center of fear (Fanselow and Poulos, 2005; Lang and Davis, 2006; Panksepp et al., 2011; Adolphs, 2013; Tovote et al., 2015). Using fMRI, Ochsner et al. (2002) showed that cognitive reappraisal to aversive images involves prefrontal and cingulate control systems that reduce amygdala activation. However, fear and anxiety experiences do not always align with behavioral and physiological responses, which suggests that fear may not solely originate from the amygdala (LeDoux and Pine, 2016). Individuals with amygdala damage can still experience fear and anxiety, but their ability to respond behaviorally and physiologically to threats may be compromised (Phelps, 2006; Adolphs, 2008; LeDoux and Pine, 2016). Panksepp et al. (2011) argue that fear is an aversive state of mind driven by a subcortical “FEAR” system. Even non-consciously processed threats can trigger peripheral physiological responses through increased amygdala activity, despite a lack of conscious awareness and feelings of fear (LeDoux and Pine, 2016).

LeDoux and Pine (2016) proposed a “two-systems” framework for understanding the neural basis of emotion, where one system is responsible for generating conscious feelings, while the other controls behavioral and physiological responses to threats. According to higher-order theories of emotion, subjective experiences are generated by more limited circuits, primarily involving a prefrontal hub that supports thoughts about lower-order information (LeDoux and Pine, 2016; LeDoux, 2021). The dual-systems approach not only distinguishes between the two circuits but also reveals that the construction of the interaction between stimulus input and the prefrontal cortex, where the mental model is located, depends on low-order memory based on semantics and scenarios and the middle-order prefrontal cortex where the schema is located. The formation of schemata requires the construction and interaction of low and intermediate levels as a basis. The dual system in emotion regulation can be likened to the relationship between the top-down central and local systems. In response to external stimuli, the local system can either act based on previous models or report to the central system. Some incentives can be addressed at the local level, while signals from the central system can be effectively implemented, often due to adaptation to local realities or resolution of the emergency. However, simply issuing instructions or only interpreting local threats and stimuli without addressing the fundamental issue may not effectively solve the problem.

As a result, there exist two different theoretical models of treatment strategies based on distinct understandings of the brain mechanisms underlying cognitive reappraisal, namely the top-down Cognitive Emotion Regulation (CER) model and the bottom-up Experiential-Dynamic Emotion Regulation (EDER) model.

Therapeutic approaches based on the Cognitive Emotion Regulation (CER) model, proposed by Gross (1998b,2014), posit that conscious cognitive appraisal is the source of emotion production and that effective emotion regulation occurs through the use of top-down regulatory strategies at various levels. These strategies include active cognitive regulation techniques such as situation selection and modification, which are aimed at down-regulating emotions. To use an analogy, top-down regulation is like issuing instructions from a central system, however, when the use of regulation strategies fails, for example in the case of cognitive dissonance, the supervisory role of the local system needs to adapted to the actual situation, in which case top-down cognitive adjustment and supervision requires new cognition to adapt to the client’s actual situation, and corresponding low-level resource adaptation and matching fundamental. The biological mechanism behind regulation is inhibitions, which involves top-down inhibition of the amygdala in the prefrontal lobe, and every emotion is either up-regulated or down-regulated. The therapeutic process is characterized by its top-down, voluntary, external, conscious, and verbally mediated features, with the neural basis of the CER approach mainly cortical and left-sided (Grecucci et al., 2020). Effective cognitive emotion regulation requires a stable and non-threatening state, with removal of threat at the amygdala level and assurance of the safety of the real environment serving as the foundation. In situations where a new stimulus is presented, local responses need to be rapidly determined based on the control ability of environmental stimuli in memory. When the intensity of the stimulus is closely related to the actual threat, the local system often needs to devote significant effort to address the problem, leaving little time for cognitive regulation of emotion.

Compared to Gross’s CER model, the Experiential-Dynamic Emotion Regulation (EDER) model (Grecucci et al., 2015, 2016; Frederickson et al., 2018) focuses on regulating emotions themselves rather than through control, by recreating real experiences that allow emotions, attitudes, and beliefs to be expressed. This approach can be likened to starting from the local actual situation. Active regulation of emotional states can be achieved by allowing oneself to experience them, focusing on bodily sensations and becoming aware of the emotional state (Vandekerckhove et al., 2011, 2012; Grecucci et al., 2020). According to the EDER model, emotions are automatically generated by attribute-specific subordinate structures, and normal emotion regulation occurs when the brain self-regulates emotions through biological mechanisms to return them to a baseline state. Dysregulation occurs when self-regulation ceases after the emotion has been generated due to interference with dysregulatory mechanisms, resulting in the down-regulation or blocking of anxiety and defensive influences. Empirical-dynamic therapy and empirical-cognitive therapy are the treatments used in the EDER model, in which clinicians help clients to remove dysregulated mechanisms and down-regulate dysregulated emotional states. Memory remodeling forms the biological basis of the therapy. The key features of the conditioning process are that it is bottom-up, involuntary, implicit, unconscious and independent of language. The neural basis of the EDER model therapy focuses on the subcortical and right side of the brain.

Due to the dual nature of emotion regulation, we suggest that the theoretical basis for such regulation should be rooted in the Experiential-Dynamic Emotion Regulation (EDER) model, which is based on a bottom-up approach. Furthermore, top-down regulation ought to be supported by bottom-up processes that emphasize the crucial role of interaction between actions and the environment in the developmental process, as highlighted in Piaget’s theory.

### 2.2. Insights from Piaget’s schema theory of cognitive development

During bottom-up processing, individuals form schemata based on their experiences. Schemata are representations or models of the self, world, and others that encode generalizable knowledge extracted from regularities across past experiences (Dalgleish and Hitchcock, 2023). A schema represents the developmental norm of cognitive structures and is formed by an individual in the process of interacting with the environment, selectively organizing the ongoing experience of each individual into subjectively meaningful patterns (Adekoya, 2013; Yin et al., 2022).

In Piaget’s theory of cognitive development, the organism engages in the process of equilibration, which involves a cycle of disequilibrium and subsequent rebalancing, in order to assimilate and accommodate to both internal and external factors. The critical aspect of this process is the interaction between the organism’s actions and the environment. These actions encompass physical actions at the level of perception and movement, as well as mental operations such as those involved in concrete and formal reasoning (Piaget, 1979; Jiang and Li, 2020). According to Piaget’s genetic epistemology, the formation of adaptive behavior schemata is contingent upon the interaction of the individual with the real-world environment, which provides feedback that can consolidate newly formed behaviors. This direct interaction with the object world is more effective in establishing causality and enacting changes in cognitive structures than observational learning or linguistic transmission of others’ experiences (Piaget and Duckworth, 1970; Yin et al., 2022).

The argument posits that the processing of information is guided by a set of active schemata that harbor negative generalizable knowledge about the self, the world, and others, which are accumulated through previous life experiences. Unlike mentally healthy individuals, those with mental health problems tend to have Schemata that encode negative content that extends further up the schema hierarchy, encompassing more abstracted negative information relevant to a wider range of contexts. This indicates that negative schematic content has become increasingly abstracted over time as individuals encounter the same negative themes across different situations (Dalgleish and Hitchcock, 2023). At higher levels, summary memories reflect lifetime periods and autobiographical themes, while at the lowest level, specific episodic records of individual events are recorded. Processing is driven by active generalized negative self-schemata that represent relatively abstracted conceptualizations of the self that are relevant to multiple life contexts. Individuals with emotional problems tend to exhibit high levels of overactive negative schemata about themselves, such as perceiving themselves as unlovable. They selectively process information from the past to be consistent with the negative self-schema, with negative autobiographical recollections being more likely to be preferentially accessed and having more complex emotional diversity relative to positive recollections. Furthermore, there is often a significant lack of positive memory experiences that can be recalled (Hallford et al., 2021).

At a high level of cognition, conversation-based cognition or communication alone is often inadequate due to the absence of fundamental processes that underpin it. The interaction between “action” and the environment is more fundamental. From an evolutionary and developmental standpoint, there is a continuum in the relationship between behavior and environmental stimuli. Addressing emotional problems at their root involves addressing the problem of mismatch between the schemata and the environment.

### 2.3. Insights from how autobiographical memory is formed and reorganized into schemata

Memory consolidation and schema development are vital components of autobiographical memory, which plays a critical role in fostering self-awareness, connecting individuals with others and the broader world, and reconstructing memories (Pillemer, 1998; Wang, 2013; Alea and Wang, 2015). Recent research has demonstrated that replay serves a crucial function in memory consolidation, schema development, and generalization, indicating that it is more than just a repetition of past experiences (Xue, 2022). Specifically, replay has been shown to have a planning function, allowing for the quick simulation of tracks in cognitive space through compressed playback (Arnold et al., 2016). Moreover, this compression-repetition enables cell pairs to co-activate within a time window that facilitates the induction of synaptic plasticity, increasing the connection between co-activated neurons (Bi and Poo, 1998; King et al., 1999; Xue, 2022).

The existence of the coincidence time window has implications for associative learning and how the brain estimates the relationship between two temporally distinct events, particularly in the context of aversive memory. The CS and US must follow a specific order and arrive in the coincidence time window, typically on the order of tens of seconds, for aversive memory to be elicited (Tully and Quinn, 1985; Tanimoto et al., 2004; Tomchik and Davis, 2009; Gerber et al., 2019; Zeng et al., 2023). The continuity of stimulus-response is important for constructing relationships between events, with scenario-based experiences forming more robust connections on the neural circuit compared to conversation-based cognitive reappraisal, which relies more on second and third level CS based on symbols, which can be challenging for inexperienced individuals to learn.

On the other hand, Memory representation can undergo reorganization, identification, integration, and extraction of the gist to form a schema. The reorganization of memory fragments is a reconstruction process aimed at aiding the transition of episodic memory into long-term memory. During this process, uncertain information enters an unstable state when reactivated, allowing for its integration with other related information and consolidation into a more stable representation (Xue, 2022). Interestingly, individuals who are flourishing are expected to exhibit reversed patterns of memory distortions, such as positive memory biases, richly recollected positive memories, unbidden positive recollections, and overgeneral positive memories. Recent research has shown that the mediation of hippocampal-based non-associative formation and striatal-based mental reward can regulate emotions, leading to novel and positive emotional experiences that can be preserved in long-term memory (Yao et al., 2022). By coupling memories with positive emotional experiences, individuals are more likely to form consolidated, positive self-schema. These consolidated schemata can, in turn, enhance the generalization of positive experiences, leading to more adaptive coping strategies and overall improved mental health outcomes (Dalgleish and Hitchcock, 2023).

## 3. A new understanding: Schema updating/enrichment may be crucial for the efficacy of the cognitive reappraisal technique

**FIGURE 1:**
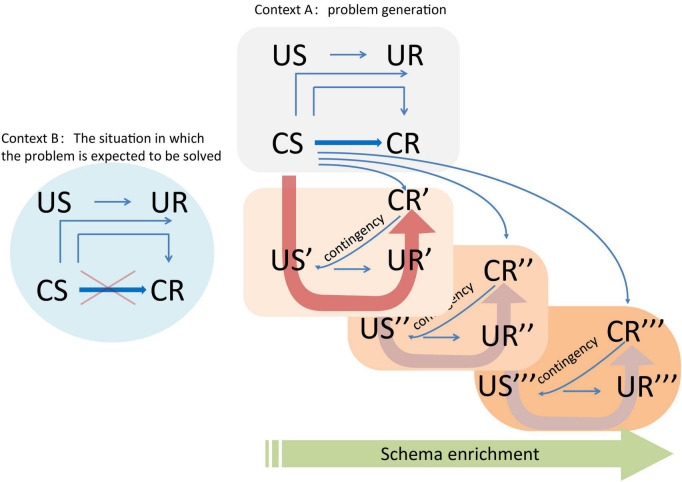
Schema-enrichment-based cognitive reappraisal technique. Context A represents the situation in which the original emotional problem arises. On the other hand, and Context B is the setting where the problem is expected to be resolved. The unconditioned stimulus (US) is a stimulus that holds significance for the survival and reproduction of individuals. The unconditioned response (UR) refers to an innate instinctive response that is triggered by the US. The conditioned stimulus (CS) is a neutral stimulus that consistently accompanies the US and trigger an individual’s learned response known as the conditioned response (CR), which functionally replaces the UR when the individual learns that the occurrence of the CS predicts the occurrence of the US. Contingency refers to the “if-then” relationship between two events (Bouton, 2016) and involves the probability of the occurrence of an US when a CS is present (Battaglia et al., 2020). The connections between the different elements are represented by blue arrows in the figure, where thick blue arrows depict higher order paths and thin arrows represent lower order paths. The red crosses indicate updates to path connections where the original path is irrelevant in the current situation. The background with different colors represents changes in scenarios, and the heavy arrow indicates the formation of a new connection (new schema). The green arrow signifies the enrichment of the schema resulting from the new connection formed during the training process, during which new CRs (annotated with strokes) lead to new USs and thus new URs.

Drawing upon the aforementioned theories and findings, here we present new insights into the practice of cognitive reappraisal. The reframing of the cognitive reappraisal technique is depicted in Figure 1. The client has learned the association between certain stimuli and negative experiences, which triggers corresponding negative emotions and responses. In the past, cognitive reappraisal techniques were applied to regulate emotions in new contexts, such as the consulting room, and only impacted higher-order processing. However, these interventions were implemented by learning new connections that were unrelated to the original context. This approach often neglected the original connections from other pathways, allowing related elements to be activated again in similar situations. Instead, the new approach emphasizes that training shall facilitate a return to the original situation and strengthens it through the promotion of beneficial behaviors and timely feedback. This helps clients gain experience in their interaction with the environment, building new connections and forming a new schema at the basic level. An enriched schema pool can activate more relevant schemata in similar situations, leading to positive emotional experiences and promoting inclusivity in the client’s interaction with the real environment.

### 3.1. The construction, updating, and enrichment of schemata

Drawing on schema theory and principles of learning behavior, the new approach is to establish a rich schema network through training. Rather than learning in a new environment, the new approach involves returning to the original situation that contains key elements, providing clients with new, timely, and positive feedback on their behaviors, and establishing various CS-US (- CR-UR) causal links. By doing so, the new approach aim to facilitate the formation of new CRs and enable clients to experience the changes brought about by these new CRs. This approach provides clients with a diverse and rich set of activated schemata that they can draw upon when encountering new CS or US, enabling them to activate different causal relationships than those previously learned and generate novel experiences. Importantly, this type of learning can only occur through personal experience (Piaget and Duckworth, 1970).

To enhance the effectiveness of cognitive reappraisal techniques, a new approach involves constructing rich scenarios that help clients build novel schemata. Rather than directly altering conversation-based cognition or communication, this approach aims to assist clients in reconstructing their environment and providing cues that effectively activate the new schema (Yin et al., 2022).

It is important to note that negative schemata can be reinforced in response to negative stimuli, leading to biases in negative emotional experiences (Disner et al., 2011). In cases of depression, cognitive vulnerability to early adverse life events and underlying depressive schemata can impair information acquisition, memory retrieval, and information processing (Hayes and Hofmann, 2018). Therefore, cognitive reappraisal may be more effective if based on enriched schemata, which serve as the foundation for triggering diverse behaviors and matching the environment to produce stable emotions. In other words, updated/enriched schemata are essential for clients to avoid activating the amygdala response when facing new stimuli, sort out existing memories, and make choices among various schemata—this is when the efficacy of cognitive reappraisal comes in.

### 3.2. Practical applications of the new approach

To summarize our previous chapters, recent research in schema theory and neuroscience suggests that effective cognitive reappraisal requires appropriate schema activation and a safe environment. However, implementing conversation-based cognitive reappraisal can be complex, and individuals may struggle to comprehend and implement it. Importantly, directly changing conversation-based interpretation or dialog may not necessarily lead to efficacious cognitive reappraisal. Instead, clients need help to restructure the context of their emotional experience and to provide cues that activate new schemata. To address this issue, enriching the client’s experiential schema is essential. This can enable the client to activate an appropriate part of their self-schema in response to environmental stimuli, leading to emotional regulation and a new balance with the environment. Enriched schema can help individuals activate positive autobiographical memories that match the environment when faced with emotions. This can then better facilitate cognitive reappraisal techniques when guided by rich schemata of multiple contextual constructs.

Returning to Lisa’s example, the therapist can tell Lisa that “maybe your friend’s expectant mother party can only be shared with important family members” so that you were not invited does not necessarily mean that “your friend doesn’t like you.” However, these conversations do not address the feeling of loss or the unfulfilled need that Lisa is experiencing. Schema enrichment training can help clients view the same context and stimuli from different angles, making it easier for them to choose dimensions of themselves that better match the current context and experience positive emotions. In Lisa’s case, by softly guiding her to engage in meaningful activities and building positive experiences, Lisa can enhance her self-worth and satisfaction, which can contribute to her emotional wellbeing.

The emerging concept of the metaverse also has the potential to facilitate emotional regulation by creating immersive scenarios that simulate emotional states and allow individuals to update their emotional experiences (Yin et al., 2022). Metaverse-based training can also be beneficial for Lisa by helping her build scenarios of important moments based on contextual cues. This can trigger various behaviors and provide timely feedback to facilitate the formation of rich schemata. Ultimately, schema enrichment can be a useful tool in helping individuals like Lisa overcome negative emotions and develop positive coping strategies for a more fulfilling life.

In conclusion, traditional-form cognitive reappraisal and schema-enrichment-based cognitive reappraisal can be complementary techniques for emotional regulation. While the former focuses on altering the interpretation of negative emotions and past experiences, the latter provides clients with multidimensional experiences that allow them to see themselves and their experiences from different perspectives. Enriching schema allows individuals to explore multiple pathways for triggering positive emotional states and generating stable emotions in challenging situations, leading to a shift in the automated cognitive reappraisal process. The richness of the schema model can then transform an individual’s behavioral choices in response to varying scenarios, leading to better matching and balance with the environment.

### 3.3. Ethical considerations of the new approach

To ensure that schema-enrichment-based cognitive reappraisal training is conducted in a responsible and ethical manner, it is crucial for the therapist to obtain informed consent from the client before starting the training. This entails ensuring that the client fully comprehends the purpose, risks, and benefits of the techniques being employed, as well as their right to terminate and withdraw from the training at any point.

Throughout the training process, the therapist must identify the key negative stimulation CS and the original environmental elements that may trigger negative emotions or memories. Good behavioral guidance and reinforcement should be utilized to assist clients in forming connections between CS and new US triggers. It is important to avoid causing secondary injuries by exposing clients to similar situations at the beginning of training. Effective communication and feedback channels are essential during the training process. If training is conducted online or in a metaverse, data analysis should be used to track changes in the client’s needs and to facilitate progress through the stages of training. Data security measures should also be implemented to prevent tampering and protect clients’ privacy.

When developing the training program, the therapist should consider individual differences in cultural background, language, and emotional objectives. Feedback on new behaviors should be customized to the client’s values and implemented based on an assessment of the client’s progress. Practitioners in the field of counseling should be vigilant about professionalism, attention to detail, and potential learning and interference. For clients with severe mental illness, a focused assessment of the applicability of emotional regulation to their condition should be conducted before starting the training. For children and teenagers, obtaining guardianship consent and providing active guidance and prevention of addiction should be prioritized. Overall, ethical considerations and responsible practice should be at the forefront of all schema-enrichment-based cognitive reappraisal training programs.

## 4. Prospects for the future

There are several potential future prospects for the use of schema-enrichment-based cognitive reappraisal training. One such prospect is the integration of emerging technologies. As technology continues to advance, there may be new ways to integrate schema enrichment and cognitive reappraisal training with virtual reality, artificial intelligence, and other emerging technologies. This could lead to more immersive and personalized training experiences, as well as better data analysis and monitoring capabilities.

There could be a shift toward preventative care and early intervention if the new approach is adopted. Instead of waiting for individuals to develop negative emotional patterns and seeking treatment, schema enrichment and cognitive reappraisal training could be incorporated into educational and workplace settings to promote emotional wellbeing and prevent the development of negative patterns. Additionally, such training could be integrated with the current form of cognitive-behavioral therapy and mindfulness-based therapy. This could lead to more comprehensive and personalized treatment plans for individuals struggling with emotional regulation and other mental health issues.

Finally, further research and validation is needed to fully understand the potential of schema-enrichment-based cognitive reappraisal training. This could involve large-scale clinical trials, longitudinal studies, and the development of standardized protocols for training and assessment across different populations and contexts. With continued research and development, schema-enrichment-based cognitive reappraisal training has the potential to revolutionize the way we approach emotional regulation and mental health treatment.

## 5. Conclusion

To summarize, there are differences between the original problematic and therapeutic contexts, and the reappraisal that occurs in the therapeutic context represents a new learning experience that may not be easily transferred to the problematic situations. Moreover, unmet psychological needs of the client may persist, necessitating multiple repetitions of reappraisal outside of safe environments such as the counseling room. The conversation-based cognitive reappraisal process is an interpretation of the behavioral process that uses the counselor to facilitate meaningful labeling of behavior and emotions. This process aids in bringing cognition back to an explainable or “dissonance” state, which matches cognition to behavior without necessarily altering behavior itself. While cognitive reappraisal is considered a technique for regulating emotions, this paper argues that schemata are more critical in guiding behavior. Matching an individual’s schemata to the current context and identifying solutions to current problems are key factors in addressing emotional problems. Enriching experiences and developing schemata at a fundamental level can provide an effective solution for underlying causes of persistent emotional disturbances and the limited effectiveness of reappraisal in non-therapeutic environments. Based on the two-system theory, reconstruction of memory and schema-related research, this paper proposes a new insight into cognitive reappraisal techniques: the possibility of creating new behaviors and outcomes based on schema enrichment and updating. With positive feedback and continuous practice, these new cause-and-effect schemata can guide the client’s behavior and lead to fundamental reappraisal effects that promote emotional stability. Last but not least, this suggests a new avenue for exploring the relationship between emotion, cognition, and behavior.

## Data availability statement

The original contributions presented in this study are included in the article/supplementary material, further inquiries can be directed to the corresponding author.

## Author contributions

Y-XW and BY conceived of the presented idea. Y-XW wrote the first draft of the manuscript. BY critically revised the manuscript. Both authors discussed and contributed to the final manuscript, and approved the final version of the manuscript.
